# Megakaryocytic Differentiation of K562 Cells Induced by PMA Reduced the Activity of Respiratory Chain Complex IV

**DOI:** 10.1371/journal.pone.0096246

**Published:** 2014-05-09

**Authors:** Rui Huang, Long Zhao, Hui Chen, Rong-Hua Yin, Chang-Yan Li, Yi-Qun Zhan, Jian-Hong Zhang, Chang-hui Ge, Miao Yu, Xiao-Ming Yang

**Affiliations:** 1 Beijing Institute of Radiation Medicine, Beijing, China; 2 State Key Laboratory of Proteomics, Beijing Proteome Research Center, Beijing Institute of Radiation Medicine, Beijing, China; Mayo Clinic, United States of America

## Abstract

Mitochondria are involved in the regulation of cell differentiation processes, but its function changes and molecular mechanisms are not yet clear. In this study, we found that mitochondrial functions changed obviously when K562 cells were induced to megakaryocytic differentiation by phorbol 12-myristate 13-acetate (PMA). During the cell differentiation, the reactive oxygen species (ROS) level was increased, mitochondrial membrane potential declined and respiratory chain complex IV activity was decreased. Treatment with specific inhibitor of mitochondrial respiratory chain complex IV led to a significant inhibition in mitochondrial membrane potential and reduction of PMA-induced cell differentiation. However, treatment with cyclosporine A, a stabilization reagent of mitochondrial membrane potential, did not improve the down-regulation of mitochondrial respiratory chain complex IV induced by PMA. Furthermore, we found that the level of the complex IV core subunit COX3 and mitochondrial transport-related proteins Tim9 and Tim10 were decreased during the differentiation of K562 cells induced by PMA, suggesting an important role of these factors in mitochondrial functional changes. Our results suggest that changes in mitochondrial functions are involved in the PMA-induced K562 cell differentiation process, and the maintenance of the steady-state of mitochondrial functions plays a critical role in the regulation of cell differentiation.

## Introduction

The mitochondria perform three important functions in the cells: energy production, execution and amplification of cell death pathways, and signal pathway regulation. Although ATP production is a key function of the mitochondria, a large number of accumulated evidences suggested that mitochondria are also involved in the regulation of cell differentiation process. Mitochondria play an important role in the physiological status maintenance and differentiation of stem cells [1], and are involved in the regulation of the differentiation of fat cells [2] muscle cells [3], nerve cells [4], and osteoblasts [5]. The inhibition of mitochondrial protein synthesis affect the differentiation of a variety of cells, including mouse erythroleukemia [6] and mastocytoma cells [7], neurons [8], and human [9], avian [10], or murine myoblasts [11]. The differentiation of oligodendroglial induces mitochondrial gene expression and inhibits mitochondrial functions [12]. In addition, recent evidence has indicated that respiratory chain complexes can be organized in a larger supercomplex in the mitochondrial inner membrane [13]. The mitochondrial super complex will undergo changes in the differentiation processes of a variety of cells [14].

Phorbol 12-myristate 13-acetate (PMA)-induced megakaryocytic differentiation of K562 cells is a classic model to study the differentiation of blood cells. This process is accompanied by the changes in cell morphology, the acquisition of adhesion properties, cell growth arrest, specific markers expressing on the cell surface of megakaryocytes and other changes [15], [16]. PMA is a PKC activator, PKC activation can activate the downstream MEK/MAPK pathway and plays a critical role in the regulation of megakaryocyte differentiation; treatment of K562 cells with PMA up-regulate jun/fos, egr and other transcription factors, and it also increase the expression of CDKIs p21^WAF1/CIP1^ and p27^KIP1^ in a p53-independent manner, thus leading to cell cycle arrest [17], [18]. The alteration in the nature of cell adhesion is related with cytoskeleton changes and integrin expression [15], [16]. In addition, it was reported that PMA also induced apoptosis in K562 cells to some extent [19]. There have been many reports on the phenotype and signaling events of PMA-induced K562 cell differentiation, but whether there are mitochondrial functional changes in the process of differentiation, and whether these changes are involved in the regulation of the differentiation process are unclear.

In this study, we found that mitochondrial functions altered significantly when K562 cells were induced to megakaryocytic differentiation by PMA. During cell differentiation, the ROS level was increased, mitochondrial membrane potential declined and respiratory chain complex IV activity was decreased. Furthermore, the protein levels of the complex IV core subunit COX3 and mitochondrial transport-related proteins Tim9 and Tim10 were decreased during PMA-induced differentiation, suggesting a potential role of these proteins in mitochondrial functional changes.

## Materials and Methods

### Cell culture and differentiation induction

Human leukemia cell line K562 was obtained from the American Type Culture Collection (ATCC, Manassas, VA) and maintained at 37 °C, 5% CO_2_ in RPMI 1640 medium (Gibco Invitrogen, CA) supplemented with 10% (v/v) fetal bovine serum.

When differentiation was induced, exponentially growing K562 cells at a concentration of 3×10^5^ cells/ml were treated with PMA (Sigma-Aldrich, St. Louis, MO).

### Assays for cell differentiation

Cell differentiation was evaluated by staining of megakaryocytic lineage marker CD41 and CD61 (eBioscience) conjugated with phycoerythrin (PE). Fluorescence signals were detected and analyzed using flow cytometry (FACScan; BD Biosciences, San Jose, CA, USA).

For morphological assessment of cell differentiation, cytospin slides were prepared with a TXD3 cell smear centrifuge and stained with Wright-Giemsa staining solutions (Baso DIAGNOSTICS INC. ZHUHAI, CHINA).

### Assays for cell apoptosis

The apoptosis was analyzed using an annexin V-FITC apoptosis detection kit (KeyGen). Briefly, cells (1×10^6^) were harvested, centrifuged at 800 g for 5 min, washed once with cold PBS, resuspended in 100 µl annexin V-FITC binding buffer, then added 5 µl annexin V-FITC and 5 µl propidiumIodide, incubated in the dark for 15 min at room temperature. Cells were then analyzed immediately by flow cytometry (FACScan; BD Biosciences, San Jose, CA, USA).

### Sample preparation for native electrophoresis

Mitochondria were isolated using a mitochondrial isolation kit (KeyGen) following the manufacturer's instructions. Then mitochondrial proteins were extracted in mitochondrial solubilization buffer (50 mM NaCl, 50 mM imidazole, 2 mM 6-amino-caproic acid, and 1 mM EDTA, pH 7.0), supplemented with 1% DM (dodecyl-maltoside) and protease inhibitor cocktail (Roche). Following the determination of protein concentration by a Bradford assay (Thermo Scientific, Rockford, IL), the protein extracts were differentially treated for BN-PAGE and hrCN-PAGE. Samples for BN-PAGE were added to BN-sample buffer [1×BisTrisACA (100 mM BisTris-HCl, 500 mM 6-amino-caproic acid, pH 7.0), 30% Glycerol, 5% Serva Blue G]. Samples for hrCN-PAGE were supplemented with hrCN-sample buffer (50% w/v glycerol, 0.1% w/v Ponceau S).

### BN-PAGE and hrCN-PAGE

BN-PAGE and hrCN-PAGE was performed according to [20], [21]. First, 5–13.5% gradient gels cast on the Bio-Rad gradient delivery system (Bio-Rad, Hercules, CA) were used. Gels was overlaid with 1×gel buffer (50 mM BisTris-HCL, 500 mM 6-amino-caproic acid, pH 7.0) and stored at 4 °C until further use. The same anode buffer (25 mM BisTris-HCl, pH 7.0) was used for BN-PAGE and hrCN-PAGE. Cathode buffer (50 mM Tricine, 15 mM BisTris-HCl, pH 7.0) containing Serva Blue G (0.02%) was for BN-PAGE; Cathode buffer (50 mM Tricine, 7.5 mM imidazole, pH 7.0) containing DOC (0.05%) and DM (0.02%) was for hrCN-PAGE. Electrophoresis was performed about 5 h at 3 mA, allowing the dye front to migrate to the bottom of the gel.

### In-gel catalytic activity assays

The in-gel assays followed the principles described by Ilka Wittig et al. [22]. The assays were performed at 20–25°C. All assays except the complex V assay (stopped by 50% methanol) were stopped using 50% methanol, 10% acetic acid fixing solution.

The complex I assay buffer: 25 mg of nitrotetrazolium blue (NTB) and 100 µl of NADH (10 mg/ml) in 10 ml of 5 mM Tris/HCl, pH 7.4. After about 3–5 min the reaction was stopped using the fixing solution.

The complex II assay buffer: 200 µl of sodium succinate (1 M), 8 µl of phenazine methosulfate (PMS, 250 mM dissolved in DMSO), and 25 mg of NTB in 10 ml of 5 mM Tris/HCl, pH 7.4. Around 10–30 min of incubation was required.

The complex III assay buffer: 5 mg of diaminobenzidine (DAB) dissolved in 10 ml of 50 mM sodium phosphate, pH 7.2. The reaction takes about 60 min of staining.

The complex IV assay buffer: the complex III assay buffer and 100 µl of horse heart cytochrome *c* (5 mM). The complex IV assay requires about 30 min.

The complex V assay buffer: preincubated 30 min in 35 mM Tris, 270 mM glycine, pH 8.3 (25 °C) containing or not containing the complex V inhibitor oligomycin (5 g/ml). Following removal of the preincubation solution, the gels were incubated in assay buffer: 35 mM Tris, 270 mM glycine, 14 mM MgSO4, 8 mM ATP, pH 8.3, containing or not containing 5 g/ml oligomycin.

### Spectrophotometric Measurement of Complex IV Activity

Mitochondrial was prepared as above described, and Complex IV was determined by following the oxidation of reduced cytochrome c at 550 nm, with 540 nm as the reference wave length in the presence of n-dodecyl-b-D-maltoside as previously described [23].

### Immunoblotting

After native electrophoresis, proteins were transferred to a polyvinylidene difluoride (PVDF) membrane (Millipore, Montreal). After electroblotting of proteins resolved by BN-PAGE, PVDF membranes were rinsed in methanol to remove Coomassie Blue. Then were blocked for 1 h in blocking buffer [20 mM Tris-HCl, pH 7.5, 137 mM NaCl, 0.05% (v/v) Tween 20 (TBST) containing 5% dried milk], and then incubated with the primary antibodies diluted in blocking buffer. The membrane was washed with TBST and then incubated with horseradish peroxidase-conjugated secondary antibodies for 1 h prior to visualization of the bands by ECL assays.

### Antibodies

All antibodies were purchased from commercial sources: anti-NDUFA9 Abs, anti-SDHB Abs, anti-UQCR2 Abs, anti-COX1 Abs, anti-COX3 Abs, anti-COX5A Abs, anti-Cyt c Abs, and anti-ATP5B Abs from MitoScience;anti-COX6A1 Abs, anti-HPO Abs, anti-Tim9 Abs, anti-Tim10 Abs, anti-Tim23 Abs, anti-IMMT Abs, and anti-GAPDH Abs from ProteinTech Grough;anti-Mia40 Abs from Abcam; anti-β-actin from Santa Cruz; CD41-phycoerythrin (PE), CD61-PE, CD11b-PE, and CD14-PE from eBioscience.

### Measurement of Mitochondrial Membrane Potential

Assay for mitochondrial membrane potential (MMP) was determined by using the fluorescent indicator JC-1 according to the manufacturer's protocol (Beyotime Institute of Biotechnology, Haimen, China). When the membrane potential is low, there is very little accumulation of JC-1 in the cells and the dye at low concentration exists as a monomer that emits green fluorescence. When the membrane potential is high, more JC-1 is accumulated and the dye at high concentration forms aggregates which cause a shift in the fluorescence emission from green to red. Thus, the emission of JC-1 can be used as a sensitive measure of membrane potential. In our experiments, cells (1×10^6^) were incubated with JC-1 working solution for 20 min at 37 °C, 5% CO_2_. Cells were then analyzed immediately by flow cytometry (FACScan; BD Biosciences, San Jose, CA, USA).

### Measurement of ROS Production

Intracellular ROS level was determined by incubating cells with 5 µM MitoSOX Red Mitochondrial Superoxide Indicator (Invitrogen, Carlsbad, CA) at 37 °C for 10 minutes. Cells were washed three times with Hank's Balanced Salt Solution (HBSS/Ca/Mg) to remove residual MitoSOX before resuspending in 300 µl 1×PBS. A flow cytometer analysis was then performed (MitoSox exciation/emission: 510/580 nm).

### Measurement of O_2_ consumption

O_2_ uptake by K562 cells during differentiation was detected using the electron spin resonance (ESP300 ESR spectrometer) and probe technique according to previous report [24]. Briefly, About 1×10^6^ K562 cells were mixed with 1×10^−4^ mol/L CTPO, sealed in the capillary, and placed in the ESR cavity. Spin probe spectra were obtained at indicated time, and then calculated the *K* parameter to determine O_2_ concentrations in solution. The incident microwave power was 1 mW, the field sweep was 5 G, and the field modulation amplitude was 0.05 G.

### Measurement of intracellular ATP content

Intracellular ATP level was measured using a luminescence ATP detection assay system (ATPlite kit, Vigorous,) according to the manufacturer's instruction. A total of 2×10^5^ cells were collected and lysed in 400 µl of 1× lysis buffer, vortex for 30 sec then centrifuge for 30 sec. Transfer 5 µl supernatant to a tube, add 50 µl Assay Reagent. The luminescence was detected with the Dual Luciferase ReporterAssay system (Promega) in a chemiluminescence analyzer (FB12 luminometer; Berthold Detection Systems).

### Measurement of Mitochondrial Mass

Mitochondrial mass was measured with nonyl acridine orange (NAO; Sigma). NAO was a fluorescent dye which specifically binds to cardiolipin in mitochondrial inner membrane. NAO staining were performed according to [25] with some modifications. Cells were incubated in PBS with 2.5 µM NAO for 10 min at 25 °C in the dark. The fluorescence intensity was measured by flow cytometry at ex/em 485 nm/535 nm.

### Measurement of Cytosolic Ca^2+^ concentration

Cytosolic Ca^2+^ concentration was determined using the calcium-sensitive fluorescent indicator Fura-3 AM as reported previously. Cells were incubated in PBS with 2.5 µM Fluo-3 AM for 30 min at 37 °C in the dark. Cells were then analyzed immediately by flow cytometry at ex/em 485 nm/525 nm (FACScan; BD Biosciences, San Jose, CA, USA).

### Confocal microscopy with NAO

After staining with NAO, cells were fixed in 4% paraformaldehyde (10 min) and permeabilzed with 0.1% Triton X-100 in PBS (10 min). Cells labeled with NAO were were acquired with ZEN 2008 imaging software on a Zeiss LSM 510 upright laser scanning confocal microscope.

### Transmission electron microscopy

Cells were fixed with a solution of 2.5% paraformaldehyde, 0.5% glutaraldehyde buffered with 0.1 M sodium phosphate (pH 7.4) for 24 h at 4 °C. Cell pellets were then embedded in Epon, as described [26]. Ultrastructural analyses were performed on a JEOL1010 electron microscope.

### Statistical analysis

All experiments were performed at least three times. Data were reported as means ±SEM and the statistical significance was assessed by one-way analysis of variance followed by Student's t-test. A value of *p*≤0.05 was considered to be significant.

## Results

### The effect of PMA-induced differentiation of K562 cells to megakaryocyte cell

**Figure 1:**
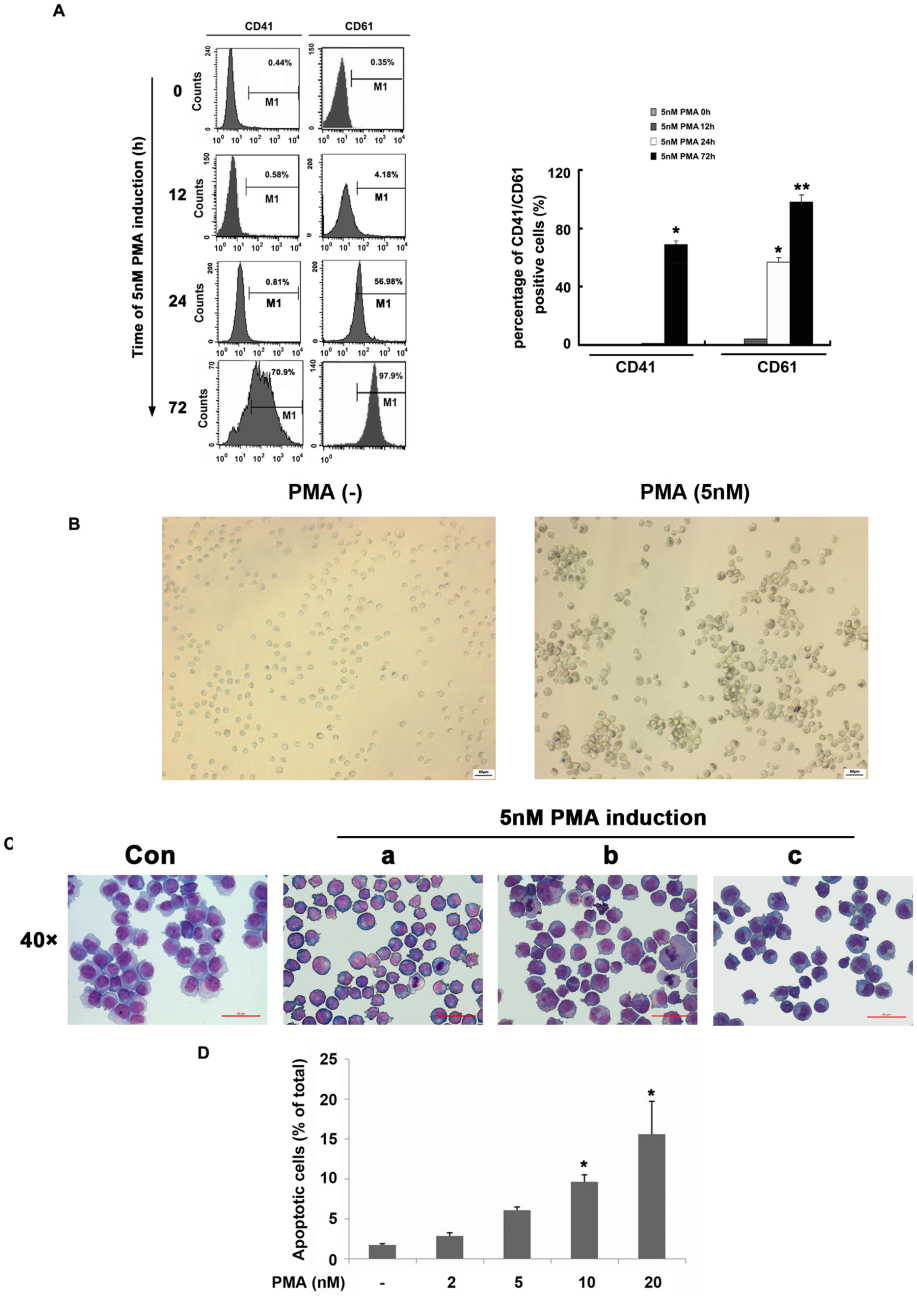
The optimization of the PMA-induced megakaryocytic differentiation in K562 cells. (A) K562 cells were incubated with 5 nM PMA for the indicated time and then the expression of CD41 and CD61 were analyzed by flow cytometry. (B) Cell morphology was observed by microscope. Scale bars represent 50 µm. (C) Megakaryocytic differentiation was detected by modified Wright-Giemsa staining for cell morphology. Representative cytological changes at 72 h, such as increase in nuclear-to-cytoplasm ratio (a), larger cells (b), and polylobulation nucleus (c) were denoted. Scale bars represent 50 µm. (D) K562 cells were induced with different concentration PMA for 72 h. Cell apoptosis were detected by flow cytometry. All graphics represented means ± SD obtained from three independent experiments, *p≤0.05, **p≤0.001.

*In vitro* megakaryocytic differentiation of the pluripotent K562 human leukemia cell line was induced by PMA. K562 cells were seeded in 6-well plates at a density of 105 cells/well in RPMI 1640 medium containing 10% fetal calf serum. Following 1 day of culture, differentiation was induced by the addition of 5 nM PMA in DMSO. Control cells received the same concentration of DMSO alone. Medium, containing PMA in DMSO or DMSO alone, was changed daily. Agreement with previously reported [27], treatment of K562 cells with PMA resulted in elevation of the expression level of CD41 and CD61 (Fig. 1A). A distinct change in morphology could be observed upon microscopic examination after 72 h induction; the cell became slightly larger and adhered stronger to each other (Fig. 1B). Wright-Giemsa staining revealed multiple megakaryocytic differentiation phenotypes such as increase in nuclear-to-cytoplasm ratio, larger cells, and polylobulation nucleus (Fig. 1C). These results indicated that 5 nM PMA-treated K562 cells were differentiated into a megakaryocytic lineage successfully.

It has been reported that PMA treatment could induce apoptosis in K562 cells [19], [27], thus we also explored apoptosis by performing FACS analysis. Consistent with previous reports, after 72 h of exposure, PMA concentrations of ≤5 nM minimally induced cell death, 10 nM PMA induced ∼10% of cells apoptosis, and 20 nM PMA induced ∼20% of cells apoptosis, indicating that PMA treatment induced K562 cells apoptosis in a dose-dependent manner (Fig. 1D). These results implied that PMA might be an effective differentiation inducer to control AML cells.

### PMA-induced megakaryocytic differentiation Attenuated Mitochondrial Activity

**Figure 2:**
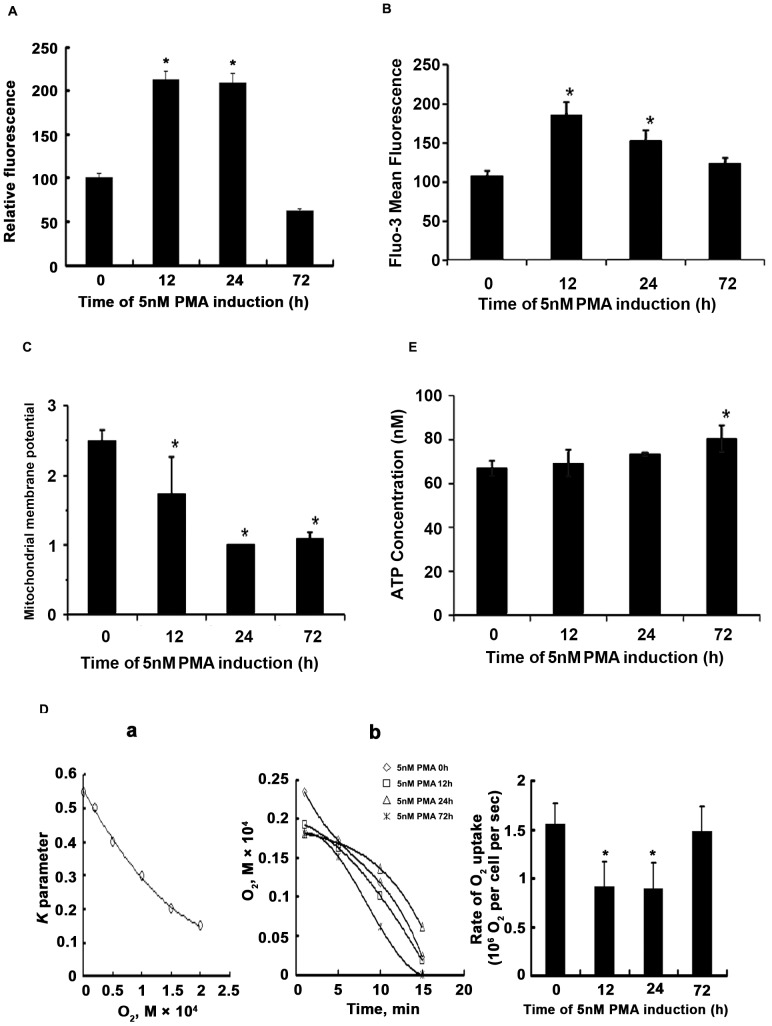
Alterations in mitochondrial functions occured during PMA-induced K562 differentiation. 1×10^6^ K562 cells were incubated with 5 nM PMA and harvested at 12 h, 24 h and 72 h respectively, stained with ROS fluorescence indicator MitoSOX (A), Fluo-3AM (B), and JC-1(C), followed by flow cytometry analysis. Data represented means ± SD obtained from three independent experiments, *p≤0.05. (D) O_2_ consumption by K562 cells during megakaryocytic differentiation were analyzed by ESR. (a): The *K* parameter curve. (b): Kinetic curve of O_2_ uptake by K562 cells. Graphics represented means ± SD obtained from three independent experiments. (E) Intracellular ATP level was measured using luminescence ATP detection assay system. Data represented means ± SD obtained from three independent experiments, *p≤0.05.

As mitochondria are the major intracellular sources of ROS production and ROS has been demonstrated necessary for megakaryocytic differentiation under PMA stimulation [19], [27]–[28], we then measured the superoxide ion production by flow cytometer analysis using Mitosox as a redox-sensitive probe (a mitochondrially targeted hydroethidine derivative). As shown in Fig. 2A, at 12 h, treatment with PMA resulted in an increase in endogenous levels of mitochondrial oxidants with fold change as 220±9.7 relative to control untreated cells (set to 100). This elevation could be sustained for 24 hours, and after 72 h, the amount of ROS was returned to basal level, presumably due to the antioxidants produced by differentiating cells to keep ROS in balance. Calcium is a key regulator of mitochondrial function and ROS could stimulate Ca2+ release from the ER [29]–[31], we therefore examined the levels of Ca2+. As shown in Fig. 2B, intracellular Ca2+ concentration was elevated to about 2∼fold, and then decreased gradually and returned to basal level after 72 h, the pattern of which is consistent with ROS. As production of ROS, acute mitochondrial uptake of Ca2+ and mitochondrial permeability transition showed a close correspondence in each case [32], [33], we also examined the time course of mitochondrial membrane permeability (Δψ_m_), a useful indicator of mitochondrial activity and status. Mitochondrial staining experiment was performed using the J-aggregate forming lipophilic cation JC-1, which normally exists in solution as a monomer emitting a green fluorescence, assumes a dimeric configuration emitting red fluorescence in a reaction driven by Δψ_m_. After treatment with PMA, we observed initial small decrease at 12 h; the decline in Δψ_m_ continued up to 24 h, decreased by about 60%; at 72 h, the Δψ_m_ increased a little, the pattern of which indicated mitochondria dysfunction during differentiation (Fig. 2C). As mitochondrial Δψ_m_ is generated by the components of the electron transport chain, which consume O2 and pump protons across the mitochondrial inner membrane to produce ATP, and any failure of mitochondrial activity must decrease the amount of O2 they utilized [34], therefore we also examined the rate of oxygen consumption. As shown in Fig. 2D, in this study, the *K* parameter was used as an empirical parameter for determining O2 concentrations in solution. The maximum rate of O2 uptake of untreated K562 cells in suspension was about 1.55×106 O2 molecules per cell per sec. We found PMA-treatment decreased the maximum rate of O2 uptake to 60% of the control value at 12 h; and at 24 h, PMA-treatment resulted in a similar inhibition of the oxygen consumption; however, the oxygen consumption returned to the basal level at 72 h. Moreover, cellular ATP level was slightly increased after 72 h of induction (Fig. 2E). It is of interest to note that after 72 h of PMA-treatment, the mitochondrial membrane remained depolarization but O2 consumption returned to normal and ATP content increased a little, which may be due to reduced ATP consumption associated with decreased ROS levels, as previously suggested [34]. These results suggested mitochondrial dysfunctions during PMA-induced megarkaryocitic differentiation of K562 cells.

### Effects of PMA-induced megakaryocytic differentiation on Mitochondrial Morphology

**Figure 3:**
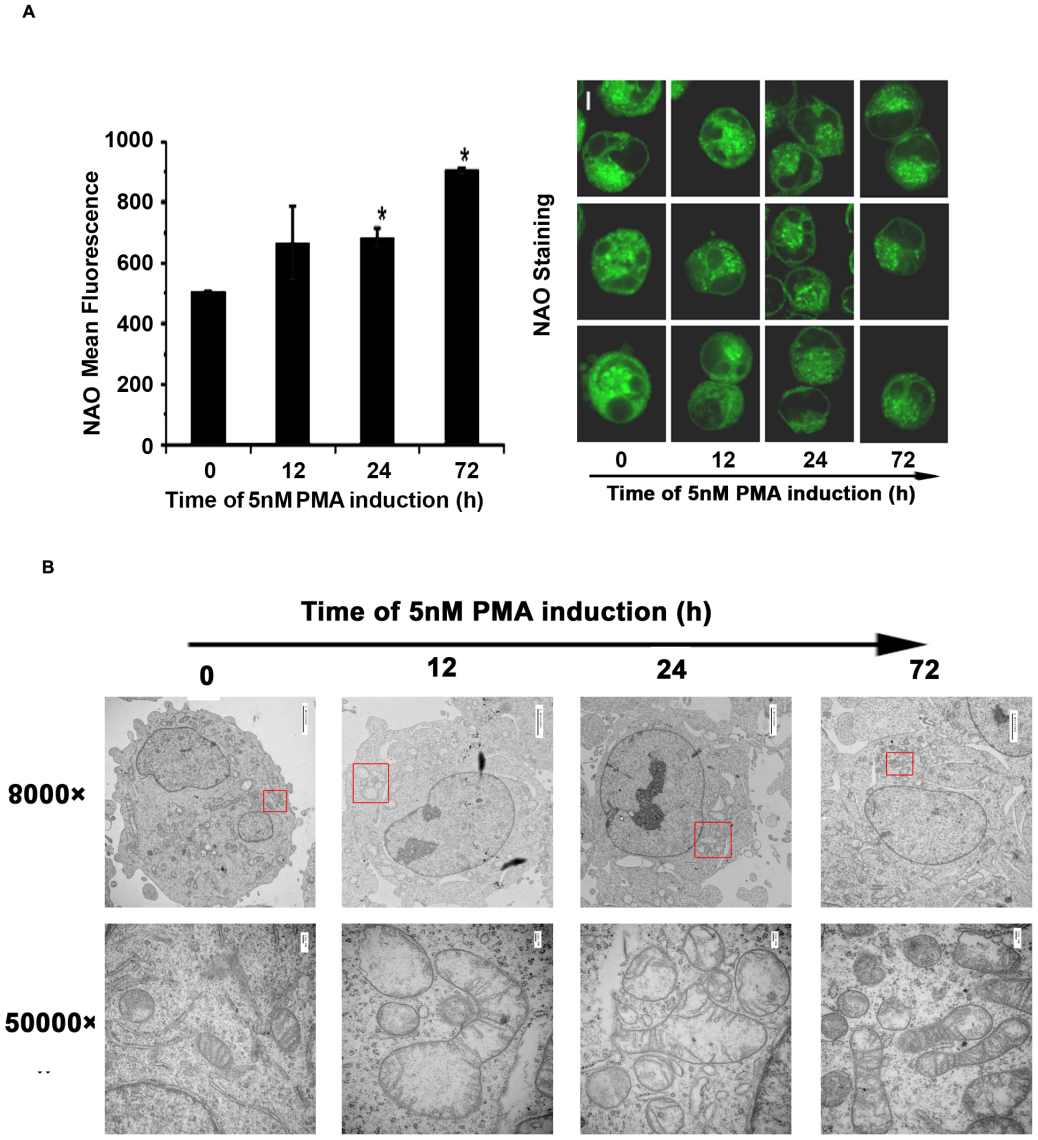
Effects of PMA-induced megakaryocytic differentiation on mitochondrial morphology and ultrastructure. 1×10^6^ K562 cells were incubated with 5 nM PMA and harvested at 12 h, 24 h and 72 h respectively, Cells labeled with NAO were detected by flow cytometry analysis and confocal microscopy (A, B). Scale bars represent 5 µm. Data were shown as mean ± SD of three independent experiments, * *p*≤0.05. (C) Mitochondria ultrastructure was analyzed by transmission electron microscopy. Scale bars represent 2 µm in the left panels and 100 nm in the right panels. The bracketed regions in the left panels are enlarged in the right panels.

To determine the effects of PMA-treatment on mitochondrial morphology, mitochondrial mass was first evaluated at different time points by staining with NAO, a fluorescent dye that specifically binds to the mitochondrial inner membrane. As shown in Fig. 3A, 5 nM PMA-treated cells showed a progressive increase in mean NAO fluorescence with time, indicating increases in the amount of mitochondrial mass during megakaryocytic differentiation of K562 cells which implied that mitochondrial fragmentation occurred after mitochondrial outer membrane permeabilization. Furthermore, we analyzed the mitochondrial ultrastructure by transmission electron microscopy. As shown in Fig. 3B, in untreated cells, small electrondense mitochondria were recognized. After 12 h of PMA induction, the mitochondria exhibited marked swelling, diminished matrix density, and disorganized cristae; similar abnormalities were observed at 24 h such as lack of cristae; however, the rearrangement of cristae occurred at 72 h although the swollen cristae and reticular cristae were also observed. As previously reported [35], the ultrastructural changes correlated with perturbation of the mitochondrial membrane permeability, which increased osmotic pressure of matrix and caused mitochondrial swelling. The cristae remodeling and fragmentation of mitochondria occurred suggested the disruption of the mitochondrial inner membrane during PMA-induced megakaryocytic differentiation.

**Figure 5:**
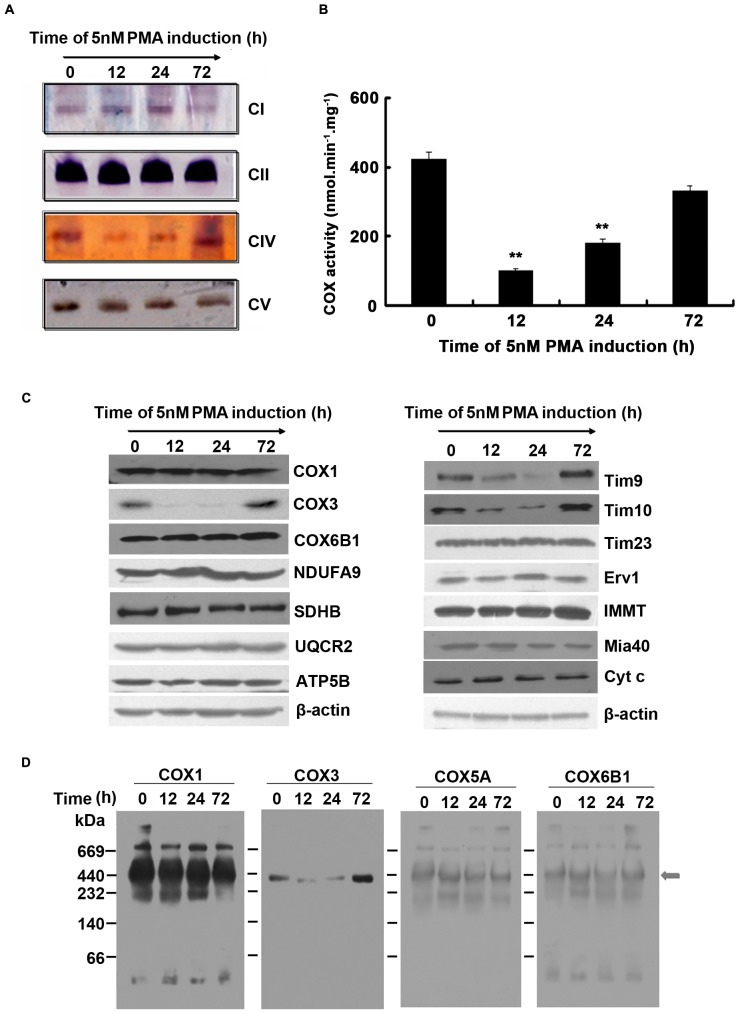
PMA induced K562 cells differentiation reduced the activity of respiratory chain complex IV. (A) K562 cells were incubated with 5 nM PMA and harvested at 12 h, 24 h and 72 h respectively. Isolated mitochondria from these cells were solubilised with 1% dodecyl-maltoside (DM) before subjecting the samples to hrCN-PAGE. After electrophoresis, gels were incubated with in-gel catalytic activity assay buffers. Each lane was loaded with 100 µg protein. (B) K562 cells were incubated with 5 nM PMA and harvested at 12 h, 24 h and 72 h respectively. The activity of the complex IV activity was detected by spectrophotometric measurement method. Data were shown as mean ± SD of three independent experiments, *p≤0.05, **p≤0.001. (C) K562 cells were incubated with 5 nM PMA for the indicated time. Mitochondrial extracts were prepared and subjected to SDS-PAGE followed by immunoblotting with antibodies indicated. (D) Mitochondrial extracts were prepared and subjected to Blue Native gel subsequently processed by immunoblotting to analyze the levels of the four subunits (COX1, COX3, COX5A and COX6B1) of complex IV. The arrows represented the complex of interest. Blots were representative of three separate experiments.

### The stability of mitochondrial membrane potential promoted the PMA-induced K562 cell differentiation

**Figure 4:**
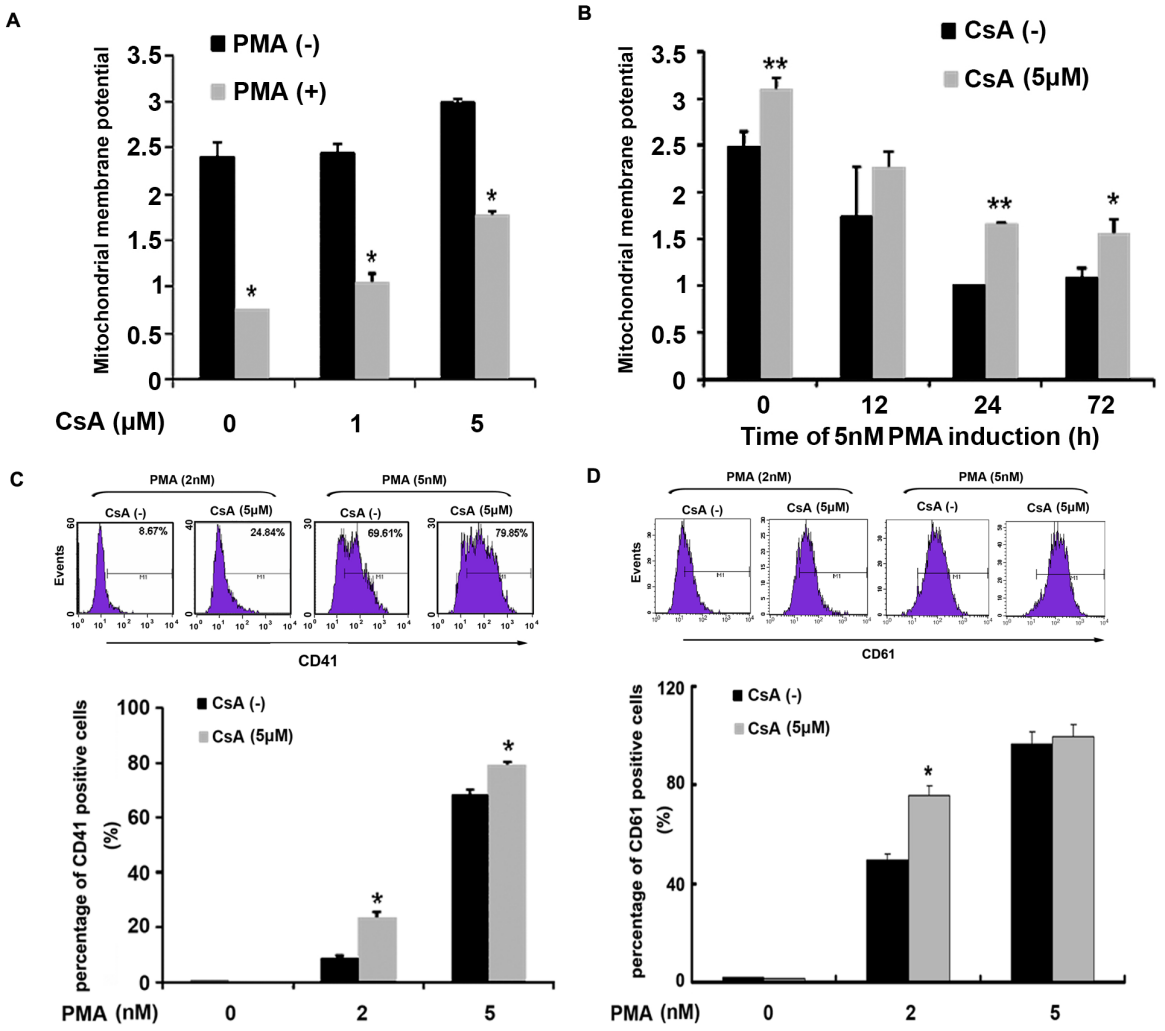
Stability of mitochondrial membrane potential promoted the PMA-induced K562 cell differentiation. (A) K562 cells were pre-treated with different doses of CsA for 6 h. After the supernatant was replaced by fresh medium without CsA, cells differentiation was induced by 5 nM PMA for 72 h. The mitochondrial membrane potential (JC-1 staining) was determined by flow cytometry analysis. (B) K562 cells were pre-treated with 5 µM CsA for 6 h, and then induced by 5 nM PMA and harvested at 12 h, 24 h and 72 h respectively. Mitochondrial membrane potential (JC-1 staining) was determined by flow cytometry analysis. (C & D) After pre-treatment by 5 µM CsA, K562 cells were induced using 2 nM or 5 nM PMA for 72 h, expression of CD41 and CD61was determined by flow cytometry analysis. All graphics represented means ± SD obtained from three independent experiments, *p≤0.05, **p≤0.001.

The stability of mitochondrial membrane potential is an indicator reflecting the mitochondrial matrix homeostasis, and it is also an important guarantee for mitochondrial to implement physiological functions. To observe the effect of the changes in mitochondrial membrane potential on PMA-induced differentiation, K562 cells were pre-treated with different concentration cyclosporine A (CsA), a mitochondrial membrane potential stabilizing agent, for 6 h, then, cells were induced by PMA. As shown in Fig. 4A and 4B, the decreasing of mitochondrial membrane potential induced by PMA was partly restored when cells were pre-treated with CsA. As shown in Fig. 4C, after 72 h of 2 nM PMA-induction, the ratio of CD41-positive cells was increased from 8.67% to 24.84% with CsA treatment; after 72 h of 5 nM PMA-induction, the ratio of CD41-positive cell ratio in CsA pre-treatment group was increased from 69.91% to 79.85%. Moreover, CD61 expression increased upon treatment as well (Fig. 4D).

### The activity of respiratory chain complex IV was reduced during megakaryocytic differentiation of K562 cells

hrCN-PAGE combined with in-gel catalytic activity assays were applied to analyze the changes of respiratory chain complexes activity during megakaryocytic differentiation of K562. As shown in Fig. 5A, PMA incubation reduced the activity of respiratory chain complex IV greatly, and the reduction recovered after 72 h. However, the activities of complex I, II, and V did not change significantly in the presence of PMA.

To confirm the reduced activity of respiratory chain complex IV by PMA treatment, we determined the COX activity of the complex IV by Spectrophotometric Measurement method. As shown in Fig. 5B, consistent with the above results, the COX activity was severely reduced at 12 h and recovered after 72 h of PMA treatment.

To study the mechanism in the repression of activity of mitochondrial complex IV during the PMA-induced differentiation of K562 cells, mitochondrial proteins were extracted, and the levels of respiratory chain complex subunits and mitochondrial protein transport related proteins were detected. As shown in Fig. 5C, after PMA induction, the expression of mitochondrial complex IV core subunit COX3 and transporter proteins Tim9 and Tim10 were significantly reduced, and there were no significant changes in the protein expressing levels of complex I subunit NDUFA9, complex II subunit SDHB, complex III subunit UQCR2, the other two subunits of complex IV including COX1 and COX6B1, and complex V subunit ATP5B.

Furthermore, BN-PAGE combined native immunoblotting analysis were adopted to detect the expression changes of the four subunits of complex IV, including COX1, COX3, COX5A and COX6B1 in PMA-induced K562 cell differentiation. As shown in Fig. 5D, COX3 expression was significantly reduced and it recovered after 72 h, which was consistent with the changes of complex IV activity. There were no significant changes in the expression levels of COX1, COX5A, and COX6B1.

### Inhibition of respiratory chain complex IV activity decreased mitochondrial membrane potential

**Figure 6:**
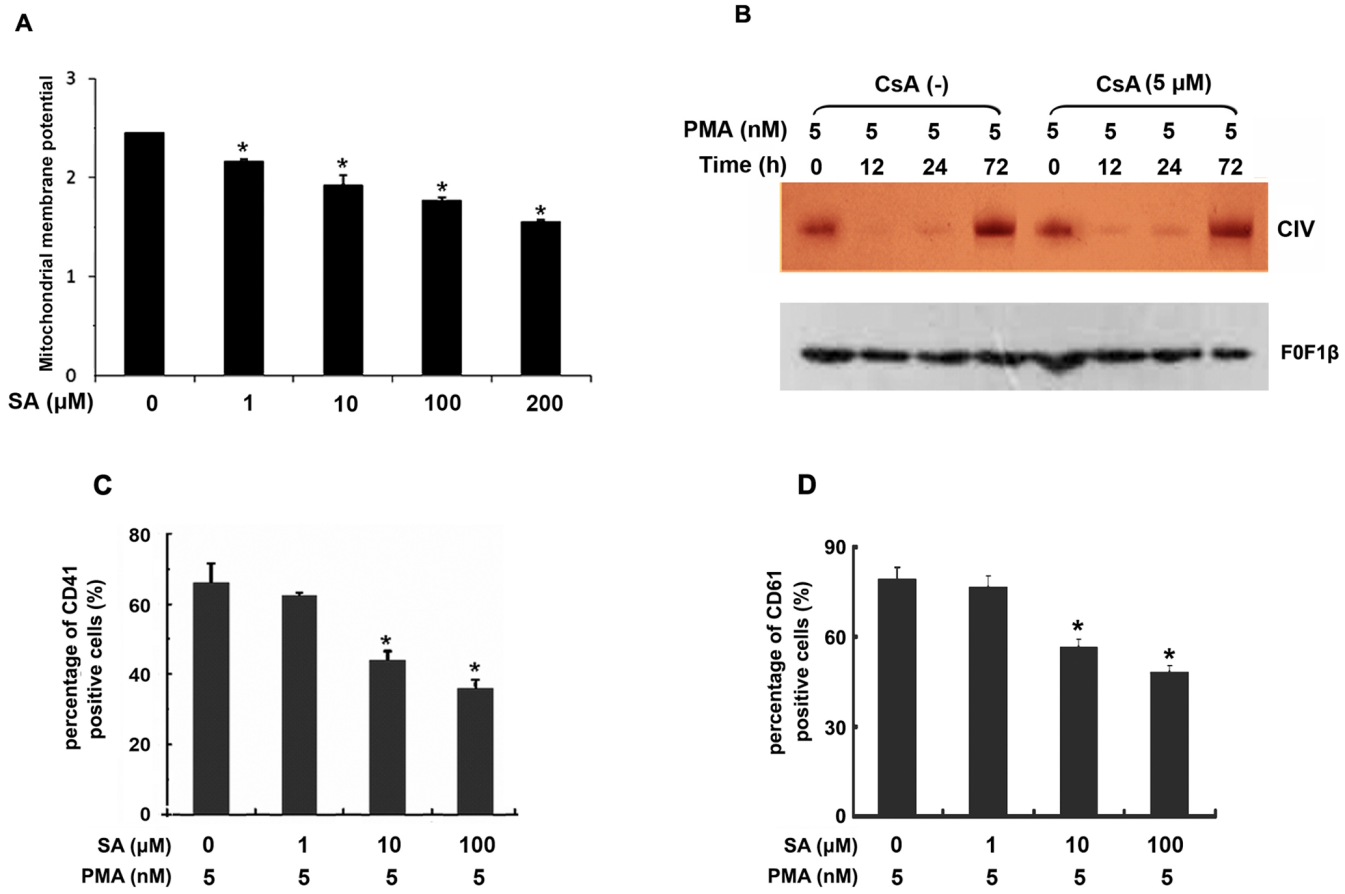
Inhibition of respiratory chain complex IV activity decreased mitochondrial membrane potential. (A) K562 cells were pre-treated with different doses of SA for 3 hour, and then the mitochondrial membrane potential (JC-1 staining) was determined by flow cytometry analysis. (B) K562 cells (3×10^5^ cells/ml) were pre-treated with 5 µM CsA for 6 h and then treated with 5 nM PMA for the indicated time. The mitochondrial protein complexes were separated by hrCN-PAGE gel and then identified by in-gel catalytic activity assay of complex IV. (C & D) K562 cells were pre-treated with the complex IV specific inhibitor SA for 3 h, then cells were induced by 5 nM PMA for 72 h. Expression of CD41 and CD61 was determined by flow cytometry analysis. Data were shown as mean ± SD of three independent experiments, *p≤0.05, **p≤0.001.

To detect the relationship between the inhibition of respiratory chain complex IV and the loss of mitochondrial membrane potential in PMA-induced K562 cell differentiation, K562 cells was incubated with different doses of complex IV specific inhibitor sodium azide (SA) for 3 h. As shown in Fig. 6A, SA reduced the mitochondrial membrane potential in a dose-dependent manner. Furthermore, pre-treatment with CsA before PMA induction to stabilize the mitochondrial membrane potential did not affect the reduction of respiratory chain complex IV activity (Fig. 6B), suggesting that during PMA-induced megakaryocytic differentiation of K562, the decreased activity of respiratory chain complex IV might be a cause, rather than a consequence, of the decrease of mitochondrial membrane potential.

To further investigate the essential role of complex IV in PMA-induced differentiation of K562 cells, we pre-treated K562 cells with the complex IV specific inhibitor sodium azide (SA) for 3 h to affect Δψ_m_, then induced cells with 5 nM PMA for 72 h, and detected CD41 expression and CD61 expression. As shown in Fig. 6C & 6D, SA treatment significantly decreased the ratio of CD41- and CD61-positive cell in a dose-dependent manner. These results suggested that complex IV involved in megakaryocytic differentiation of K562 cells induced by PMA through regulating mitochondrial membrane potential.

## Discussion

In this study, we reported that the changes in mitochondrial functions were involved in the megararyocytic differentiation of K562 cells indued by PMA, PMA-induced the activity decline in mitochondrial respiratory chain complex IV, thereby leading to changes in mitochondrial membrane potential etc. and might be the key factors leading to mitochondrial functional changes.

Leukemia cells usually have respiratory chains with higher activities; they maintain their high levels of ATP based on oxidative phosphorylation [20]. In the process of terminal differentiation, the cells acquire the metabolic characteristics of differentiation mature cells through partial loss of respiratory chain complex activities [21]. We found that PMA treatment of K562 cells induced significant activity decline in complex IV, which correlated with the low O_2_ consumption and might be a change by the cells in order to meet the metabolic characteristics of differentiated cells. On the other hand, PMA not only induce cell differentiation, but also induce apoptosis to some extent [19], [27]. In PMA-induced differentiation of K562 cells, cell growth arrest occurred, with increasing doses of PMA, the proportion of apoptotic cells significantly increased. Mitochondrial damages are involved in apoptosis [36], and whether the changes in mitochondrial functions were involved in PMA-induced K562 cell differentiation process is under further research. In rat oligodendrocytes, the inhibition of mitochondrial complex IV lead to mitochondrial damages and affect cell differentiation [37]. In fact, we also found that the inhibition of the respiratory chain complex IV activity with specific inhibitors could reduce PMA-induced differentiation.

Previous studies have shown that changes in complex activity could affect mitochondrial membrane potential. Through the inhibition of respiratory chain complex I, TNF induce cytochrome *c* release, mitochondrial membrane depolarization (membrane permeability transition) and the occurrence of apoptosis [38]. In human neutrophils, complex III play a key role in the maintenance of mitochondrial membrane potential [14]. We found that treatment of K562 cells with complex IV inhibitor resulted in significant reduction of mitochondrial membrane potential, suggesting that the PMA-induced decline in complex IV activity might be one of important reasons leading to the decline of mitochondrial membrane potential. The stability of mitochondrial membrane potential is an important guarantee for the implementation of mitochondrial physiological functions; the changes in membrane potential affect mitochondrial functions in many aspects [14], [39]. The main cause of loss of mitochondrial membrane potential is mitochondrial permeability transition (MPT) pore opening, which allows the non-selective diffusion of solutes (<1500 Da) across the membrane with resulting organelle swelling and membrane rupture [22]. As the MPT pore opening is an irreversible step in both apoptotic and necrotic cell death, it has been proposed that MPT pore opening may exist in several distinct permeability states ranging from a low conductance (allowing permeability to ions only) to a high conductance that permits translocation of a bigger molecules (<1500 Da) across mitochondrial membranes [23]. Then it would be possible that persistent, wide MPT pore opening leading to mitochondrial outer membrane rupture would cause necrotic cell death when ATP supply is not adequate; whereas transient, small amplitude MPT pore openings without cellular ATP depletion could lead to apoptosis; moreover, when the degree of mitochondrial swelling appeared to be relatively mild and did not necessarily cause a rupture of its outer membrane, the cells would remain viable [23]. In our experiments, during 5 nM PMA-induced K562 cell differentiation process, we identified the loss of Δψ, the mitochondrial swelling, mitochondrial fragmentation, and cristae remodeling without released cytochrome *c* or ATP depletion or apoptosis. These results implied that permeability states located at low conductance, which induced growth arrest rather than apoptosis. It has been reported that MPT can be reversible, but if the trigger does not cease, the Δψ became irreversible [25]. In our experiments, we demonstrated that the decrease in activity of complex IV might be one of the reasons of the decrease of Δψ; however there were low levels of mitochondrial membrane potential with normal levels activity of complex IV after 72 h of PMA treatment. This result seems inconsistency, but mitochondrial membrane permeabilization is a complex process and includes several mechanisms such as Bcl-2 family proteins regulation and lipid peroxidation, therefore the simplest explanation is that other reasons that affect Δψ might to be remained which needed further research. In recent years, works from several laboratories showed that the mitochondrial membrane potential was essential for the membrane anchorage of the respiratory supercomplexes [40], which might serve to reduce the diffusion distance of the substrates, to improve electron transfer, to decrease the reactive oxygen species formation and to stabilize the individual complexes [41]. In our study, we found that the stability of mitochondrial membrane potential promoted PMA-induced cell differentiation, possibly because of the increased stability of supercomplexes. The induction of cell terminal differentiation is an important way of leukemia therapy. Human acute promyelocytic leukemia (APL) can be treated by retinoic acid-induced differentiation and this is a successful example [42], however, the results are unsatisfactory for other types of leukemia. One reason is that the toxicity of drugs on cells and the low rate of induced differentiation. The addition of mitochondrial protective agents during the induced differentiation might maintain mitochondrial stability, or promote cell differentiation, and improve the treatment efficiency.

Complex IV consists of three core subunits encoded by mitochondrial DNA (COX1, COX2, and COX3) and 10 nuclear-encoded subunits. We found that the expression of core subunits COX3 decreased in PMA-induced cells. The down-regulation and mutations of COX3 significantly affect the activity of complex IV [43], and it is associated with many diseases [44]–[46]. In addition, the mitochondria contain about 1000 kind of proteins, more than 98% of them are encoded by nuclear DNA, and they are synthesized in the cytoplasmic ribosomes, then transported to the mitochondria and sorted and located in various parts, this process is important to maintain the steady-state mitochondrial functions. It has been reported that in the 12-O-Tetradecanoyl-1-phorbol-13-acetate (TPA) -induced differentiation of HL-60 cells, the mitochondrial transport system was down-regulated [47]. Our research found that PMA treatment down-regulate the expression levels of Tim9 and Tim10 proteins. Tim9 and Tim10 are important molecules that are located in the mitochondrial intermembrane space and involve in transport of mitochondrial targeting proteins, their down-regulation can lead to mitochondrial function disorders. Tim9 and Tim10 are small proteins with a conserved twin Cys-X_3_-Cys motif, which functions as chaperones that guide hydrophobic precursors of β-barrel proteins and carrier proteins through the IMS. The cysteine residues are essential for small Tim proteins import and assembly of the mature complexes which could not return to cytoplasm through TOM complex [48]. Thioredoxin (Trx) is a small, multi-functional protein that has a redox-active disulfide/dithiol group within the conserved active site sequence Cys-Gly-Pro-Cys. It catalyzes reduction of protein disulfide bonds and participates in folding of proteins and protein stability [49]. Moreover, it has been reported that Trx involved in mtDNA transcription through increasing the affinity of mitochondrial transcription factor A (mt-TFA) with DNA [50]. It has been reported that the decreased Trx mRNA expression was detected in the PMA-induced K562 cells [51]. Therefore, we hypothesized that the down-regulation of Tim9 and Tim10 might be due to the decreased Trx which could not form a transient disulfide bonds with Tim9 or Tim10 after they cross the TOM complex so that Tim9 and Tim10 could not assemble of the mature complexes to stay in mitochondrial intermembrane space; the down-regulation of COX3 might be due to the abnormality of Trx in mtDNA transcription regulation. The drastic restoration of complex IV, COX3, Tim9 and Tim10 regardless of CsA pre-treatment might be due to the antioxidants produced by differentiating cells to keep ROS in balance at 72 h which counteracted Trx functions. However, the exact mechanism of the down-regulation of COX3, Tim9 and Tim10 by PMA needs further study.

Complex IV (cytochrome *c* oxidase) is the terminal enzyme in the mitochondrial respiratory chain, catalyzes the electron transfer from reduced cytochrome *c* to molecular oxygen, which is reduced to water [52]. This reaction is coupled to the extrusion of protons from the mitochondrial matrix to the intermembrane space, forming a proton-based membrane potential that allows ATP to be synthesized [53]. This process is not completely efficient; electron transfer to O_2_ may occur at complex I or III, resulting in generation of reactive oxygen species, which causes cell dysfunction or death. A balance has been reported between transfer of electrons to O_2_ at complex IV to form water as opposed to premature electron transfer at complex I or complex III to form superoxide radical and inhibition mitochondrial respiration at complex IV can elevate ROS [54]. Our data also showed the correlation between the declined complex IV activity and ROS production during megakaryocytic differentiation of K562 cells. Participation of mitochondria in calcium signal has been extensively studied. Oxidants activate Ca^2+^ channels, repress pumps, and can reverse Na^+^/Ca^2+^, which cause Ca^2+^ influx into the cytoplasm [39]. In our study, the pattern of Ca^2+^ changes in the presence of PMA was considered to be due to the level of ROS increased and followed by decreasing over time. Our results showed that PMA-induced differentiation was associated with an increase in mitochondrial number but without a significant increase in mitochondrial transmembrane potential. The appearance of mitochondrial swelling has also been observed. These results suggested an accumulation of mitochondria with low transmembrane potential. Mitochondrial fragmentation may function as a compensatory response to restore an adequate mitochondrial function and lead to an accumulation of dysfunctional mitochondria.

In summary, during megakaryocytic differentiation of K562 cells induced by PMA, significant changes occurred in mitochondrial functions with declined complex IV activity and mitochondrial membrane potential and increased level of intracellular ROS. These changes might be related to the down-regulation of complex IV core subunit COX3 and mitochondrial transport proteins Tim9 and Tim10 by PMA. The stability of mitochondrial membrane potential cooperates with PMA-induced differentiation of K562 cells.
